# The method of attachment influences accelerometer-based activity data in dogs

**DOI:** 10.1186/s12917-017-0971-1

**Published:** 2017-02-10

**Authors:** Kyle W. Martin, Anastasia M. Olsen, Colleen G. Duncan, Felix M. Duerr

**Affiliations:** 1 Department of Clinical Sciences, James L. Voss Veterinary Teaching Hospital, Colorado State University, 300 W Drake Road, Fort Collins, CO 80523 USA; 2 Department of Microbiology, Immunology, and Pathology, Colorado State University Veterinary Diagnostic Laboratory, 300 W Drake Road, Fort Collins, CO 80523 USA; 3 Department of Veterinary Clinical Sciences, Purdue University College of Veterinary Medicine, 625 Harrison Street, West Lafayette, IN 47907 USA

## Abstract

**Background:**

Accelerometer-based activity monitoring is a promising new tool in veterinary medicine used to objectively assess activity levels in dogs. To date, it is unknown how device orientation, attachment method, and attachment of a leash to the collar holding an accelerometer affect canine activity data. It was our goal to evaluate whether attachment methods of accelerometers affect activity counts. Eight healthy, client-owned dogs were fitted with two identical neck collars to which two identical activity monitors were attached using six different methods of attachment. These methods of attachment evaluated the use of a protective case, positioning of the activity monitor and the tightness of attachment of the accelerometer. Lastly, the effect of leash attachment to the collar was evaluated. For trials where the effect of leash attachment to the collar was not being studied, the leash was attached to a harness. Activity data obtained from separate monitors within a given experiment were compared using Pearson correlation coefficients and across all experiments using the Kruskal-Wallis Test.

**Results:**

There was excellent correlation and low variability between activity monitors on separate collars when the leash was attached to a harness, regardless of their relative positions. There was good correlation when activity monitors were placed on the same collar regardless of orientation. There were poor correlations between activity monitors in three experiments: when the leash was fastened to the collar that held an activity monitor, when one activity monitor was housed in the protective casing, and when one activity monitor was loosely zip-tied to the collar rather than threaded on using the provided metal loop. Follow-up, pair-wise comparisons identified the correlation associated with these three methods of attachment to be statistically different from the level of correlation when monitors were placed on separate collars.

**Conclusions:**

While accelerometer-based activity monitors are useful tools to objectively assess physical activity in dogs, care must be taken when choosing a method to attach the device. The attachment of the activity monitor to the collar should utilize a second, dedicated collar that is not used for leash attachment and the attachment method should remain consistent throughout a study period.

## Background

Accelerometry has recently been introduced to veterinary medicine as a novel outcome measurement to objectively assess activity levels in dogs. This technology represents a valuable tool that has frequently been utilized in conjunction with other, previously validated outcome measures such as ground reaction forces and validated owner questionnaires [1–11]. Omnidirectional activity monitors measure spontaneous activity over an adjustable period of time (“epoch”) and filter out constant sources of acceleration (i.e. gravity) [7].

Many studies have investigated the utility of accelerometer-based activity monitors for various applications, [1–19] particularly including the measurement of activity after various therapies for dogs with osteoarthritis [1, 3, 4, 16, 18, 19]. Interestingly, there are some instances where improvement of currently accepted outcome measures (e.g. owner questionnaires, gait analysis) was not accompanied by similar improvement in accelerometer-based activity levels [4, 15, 16, 18, 19]. The reason for this disconnect may be that these outcome measures evaluate different components of improvement associated with the successful treatment of osteoarthritis or that variables affecting activity data were not controlled for. Such reported variables include signalment, [2, 10, 15] body weight, [10, 15] body conformation, [2] and activity monitor positioning [8]. However, other factors such as method of accelerometer attachment to the collar have not been investigated. While many studies describe attaching the activity monitor to the collar of study participants [2–8, 10–12, 18], only few authors have provided detailed descriptions of how the activity monitor was specifically attached [5, 7, 8, 12]. Given the high sensitivity of these devices [12], it seems possible that factors such as leash attachment to the collar and tightness of monitor attachment to the collar may affect activity data. Furthermore, the use of a protective case (to extend waterproof capabilities and protect the costly activity monitors) has been previously described, [12] but the impact of this case on activity data is unknown. To the authors’ knowledge, no studies have been conducted that investigate these factors as sources of activity data variability. Hence, it was our goal to identify how specific attachment methods might affect activity measurements. Specifically, our objective was to evaluate whether device orientation, use of a protective case, attachment of an activity monitor using zip ties, and connection of a leash to the collar holding the activity monitor would have a significant impact on total activity counts. We hypothesized that these factors would significantly affect activity data.

## Methods

### Animals

Client-owned dogs were recruited from faculty, staff, and students of the Colorado State University Veterinary Teaching Hospital. Each dog was deemed clinically healthy by owner history and thorough physical examination. The dogs were individually fitted with two identical neck collars^1^ to which two identical activity monitors^2^ were attached using six different methods of attachment as outlined below. The collars were adjusted to ensure a snug fit and the specific hole in the collar used to secure it was noted for each dog to ascertain that the amount of tension on the collar was consistent throughout the study period as previously reported [20]. Due to limitations in the ability to consistently attach activity monitors to excessively small or large collars, small and giant breed dogs were excluded from the study. The study protocol was approved by the Institutional Clinical Research Review Board (VCS#2015-029) and written owner consent was obtained.

### Activity monitoring

**Fig. 1:**
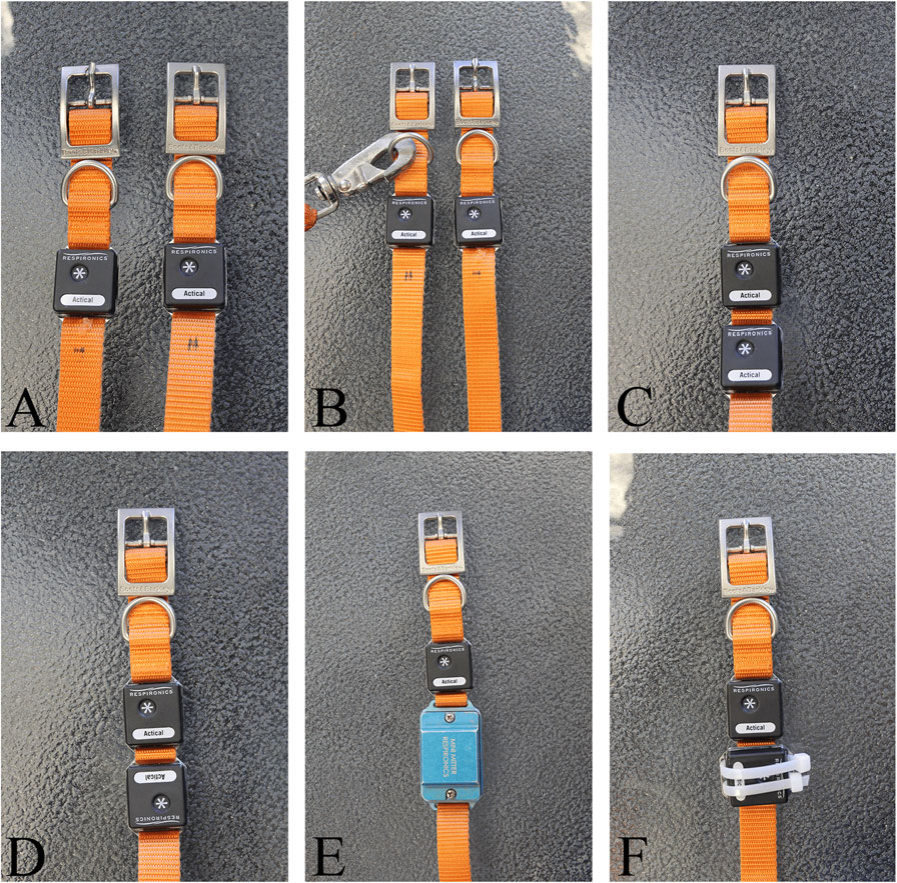
Photographs illustrating the various methods of attachment utilized in this study. **a** The activity monitors were threaded onto separate collars using the provided metal loops on the monitors. This method of attachment was utilized in Experiments 1 & 2. **b** The activity monitors were threaded onto separate collars using the provided metal loops on the monitors and a leash was attached to the rostral collar. This method of attachment was utilized in Experiment 3. **c** The activity monitors were threaded onto the same collar in the same orientation using the provided metal loops on the monitors. This method of attachment was utilized in Experiment 4. **d** The activity monitors were threaded onto the same collar in opposite orientations using the provided metal loops on the monitors. This method of attachment was utilized in Experiment 5. **e** The activity monitors were placed on the same collar. One monitor was threaded on using the provided metal loop on the monitor. The other activity monitor was placed in the same orientation as the first, but inside a metal protective case on the same collar. This method of attachment was utilized in Experiment 6. **f** One activity monitor was attached using the provided metal loop on the monitor. The other monitor was rotated 90° and attached to the same collar using zip-ties. This method of attachment was utilized in Experiment 7

A previously validated [1–11], omnidirectional accelerometer-based activity monitor was used in all dogs. Six methods of attachment were studied (Fig. 1) in seven different experiments. Eight dogs participated in the study (4 neutered males, 4 spayed females). The mean age ± SD was 3.8 ± 2.4 years (range: 1.2–8.7 years) and the mean weight ± SD was 21.3 ± 5.77 kg (range: 12.2–29.3 kg). Breeds included Border Collie (*n* = 3), mixed breed (*n* = 2), Labrador Retriever (*n* = 1), Golden Retriever (*n* = 1), and Husky (*n* = 1). Two separate accelerometers were first attached in identical fashion using the metal loops of the accelerometers to two separate, identical collars (Fig. 1a; Experiment 1). For this experiment, the leash attached to a harness^3^. Inter-collar rotation was subjectively monitored for during each data collection. If it was noted that inter-collar rotation occurred, that data set was discarded and data collection was repeated. The rostral/caudal position of these collars was then switched (Experiment 2). Next, the leash was attached to the rostral collar instead of the harness (Fig. 1b; Experiment 3). Accelerometers were then placed on the same collar in the same orientation and the second collar was removed (Fig. 1c; Experiment 4). The orientation of one accelerometer was then changed by rotating it 180° (Fig. 1d; Experiment 5). One accelerometer was then placed inside a protective case (provided by the manufacturer) with the same orientation as the other accelerometer on the same collar (Fig. 1e; Experiment 6). Finally, one accelerometer was rotated 90° and attached loosely with zip-ties to the same collar (Fig. 1f; Experiment 7). This trial necessitated the rotation of one accelerometer 90° as holes would have needed to be placed in the collar to facilitate the use of zip ties without rotation.

Three trials were collected for each dog in each experiment. A trial consisted of a 3-min outdoor leash walk across a concrete surface. The collars were oriented so that the activity monitors rested ventrally on the dog’s neck. The epoch length was set to 1 s, resulting in approximately 180 data points for every trial. The same handler walked each dog in the same fashion (i.e. the same location and speed was kept subjectively consistent). For trials where the effect of leash attachment to the collar was not being studied, the leash was attached to a harness. To mark the beginning and end of each trial, the handler pressed the event marker button located on each activity monitor. Activity data was retrieved from the monitors using the provided communications interface^4^ and software.

### Data analysis

Post-collection processing of data sets included alignment of the starting points of each data set by visual analysis within a commercially available spreadsheet software.^5^ Engagement of the event marker buttons on each accelerometer was identified within the spreadsheet program. If the data sets were not aligned, the timing was adjusted so that they were in alignment prior to statistical analysis. Statistical analysis was conducted using commercially available software.^6^ The correlation of the average activity between accelerometers was evaluated by calculating the Pearson’s correlation coefficient for each experiment. The correlation between accelerometers was compared across all experiments using a Kruskal-Wallis Test with post-hoc pairwise comparisons. Significance levels were adjusted using the Bonferroni correction.

## Results

There was no statistically significant difference (*p* > 0.05) between data from individual trials in any given experiment, thus data from all trials for each dog were included in subsequent analysis; results are summarized in Table 1. There was excellent correlation and low variability between activity monitors on separate collars when the leash was attached to a harness, regardless of their relative positions (Experiments 1 & 2, CC > 0.9). There was also good correlation when activity monitors were placed on the same collar regardless of orientation (Experiments 4 & 5, CC > 0.75). However, confidence intervals for these experiments were wider than the experiments with activity monitors on separate collars when the leash was attached to a harness. Use of the protective case, leash attachment to the collar, and attachment with zip-ties resulted in the lowest correlations between collars (CC = 0.43, 0.62, 0.64 respectively). When correlation coefficients were compared across attachment methods, there was a statistically significant difference (*p* < 0.0001) in the level of correlation in these three experiments. Follow-up, pair-wise comparisons identified the correlation associated with these three methods of attachment to be statistically different (*p* < 0.05) from the level of correlation when activity monitors were placed on separate collars.

**Table 1:** Description and correlation of attachment methods used in each experiment

Experiment	Device 1				Device 2				Mean Pearson’s Correlation Coefficient	95% Confidence Interval
	Attachment	Collars	Leash	Collar Position	Attachment	Collars	Leash	Collar position		
1	Metal Loop	Separate	Harness	Rostral	Metal Loop	Separate	Harness	Caudal	0.918	0.883–0.953
2	Metal Loop	Separate	Harness	Rostral	Metal Loop	Separate	Harness	Rostral	0.932	0.905–0.958
3	Metal Loop	Separate	Rostral Collar	Caudal	Metal Loop	Separate	Rostral Collar	Rostral	0.615	0.467–0.764
4	Metal Loop	Same	Harness		Metal Loop	Same	Harness		0.786	0.639–0.933
5	Metal Loop	Same	Harness		Metal Loop –Flipped 180°	Same	Harness		0.76	0.603–0.917
6	Metal Loop	Same	Harness		Protective Case	Same	Harness		0.428	0.217–0.638
7	Metal Loop	Same	Harness		Zip-ties – Flipped 90°	Same	Harness		0.64	0.499–0.780

## Discussion

Accelerometers have become a promising tool to objectively quantify both spontaneous and controlled physical activity in dogs. However, few studies have investigated the effect of the accelerometer positioning on the dog’s collar. Our research identified the following methods of attachment to have a significant impact on the resultant activity data: (1) attaching a leash to the dog’s collar that holds an activity monitor, (2) the use of a protective case and (3) attachment of the device loosely to the dog’s collar using zip-ties. There was high correlation between activity monitors when they were on separate collars and the leash was attached to the harness, regardless of the relative collar position. This result was expected, as identical collars were used and there were no other confounding factors in this experiment. Additionally, there was strong correlation between activity monitors when they were on the same collar, regardless of orientation. Again, this was expected, as the activity monitors used in this study are omnidirectional and record activity data in all axes, irrespective of sensor orientation.

Non-ambulatory movements of the dog have previously been suggested as factors that could affect activity data [7, 14]. Similarly, the difference in activity data found in our study associated with leash attachment to the collar can easily be explained by additional or restricted movement of the collar caused by pull of the handler/leash and a different position of the accelerometer (dorsal compared to ventral) when tension is applied to the leash. Surprisingly, the majority of previous publications do not specify whether a separate collar was used to hold the activity monitor throughout the study period (Table 2). The findings of this study suggest that a second, dedicated collar should be utilized to attach an activity monitor. It is the authors’ current practice and recommendation that the leash attachment ring (of the second, dedicated collar) should be removed to avoid any possibility of the dog’s owner attaching a leash to the collar bearing an accelerometer.

**Table 2:** Summary of methods used in previous studies with the same activity monitor

Authors	Year	Method of Attachment	Leash Attachment	Device orientation
Yam, et al.	2011	Zip tied to collar	No leash	Not addressed
Hansen, et al.	2007	Various	No leash	Not addressed
Preston, et al.	2012	Various	Not addressed	Not addressed
Yashari, et al.	2015	Protective case on dedicated collar	No leash	Not addressed
Brown, et al.	2010	To collar – no details	Leash attached	Not addressed
Brown, et al.	2010	To collar – no details	Not addressed	Not addressed
Morrison, et al.	2014	To collar – no details	Not addressed	Not addressed
Morrison, et al.	2014	To collar – no details	Not addressed	Not addressed
Rialland, et al.	2012	To collar – no details	Not addressed	Not addressed
Rialland, et al.	2013	To collar – no details	Not addressed	Not addressed
Michel, et al.	2011	To collar – no details	Not addressed	Not addressed
Dow, et al.	2009	Not addressed	Not addressed	Not addressed

The second method of attachment associated with poor correlation was when the metal protective casing provided by the manufacturer was used to house the activity monitor (Fig. 1e; Experiment 6). The accelerometer utilized in this study is currently priced at $450 and is rated for no greater than 1 meter of water submersion for 30 min. The protective case offers a simple way to extend the water-resistant capabilities of the device and protect it from incidental damage. However, our results indicate that the use of the protective case may affect activity data. These differences could be the result of the extra weight added to the activity monitor with the casing, providing it with more momentum to move on the neck. Alternatively, it is possible that the device shifts within the casing. It should also be noted that while using the protective case, the event marker buttons located on the activity monitors are inaccessible. These event marker buttons make it possible for owners to conveniently make note of significant events that occur while the dog is wearing the activity monitor, and so use of the protective casing may limit the usefulness of the monitor.

When two monitors were placed on the same collar in identical fashion (Fig. 1c & d; Experiments 4 & 5), lower than expected correlations were found between activity monitors. Furthermore, we did not find a significant difference between correlations of these experiments and when the device was placed in the protective case. This may indicate that the additional weight may play a role in accelerometer data acquisition. Based on these results, it seems that attaching two activity monitors to the same collar or adding any additional weight to the collar may affect activity data. However, further research is required to confirm these hypotheses. It should be noted that inter-device variability cannot be entirely ruled out as a cause for the low correlations found between accelerometers in Experiments 4 and 5. However, since there were strong correlations between activity monitors when they were on separate collars, inter-device variability is a less likely cause.

Finally, differences in activity data were found when the device was loosely zip-tied to the collar. When compared to the activity monitor that was threaded onto the collar using the provided metal loop, the zip-tied device was subjectively more movable. Logically, the low correlation between monitors in this experiment appears to be related to the activity monitor moving on the collar itself, artificially increasing activity counts. This occurred in spite of the zip ties being maximally tightened. These results are noteworthy because previous studies have reported zip-tying the activity monitor to the collar but did not detail the zip-tie method used [5]. Zip-tying the devices ideally should be avoided, however, given the small size of the metal loops (and thus the inability to fit thicker collars i.e. > 0.75 inches), care should be taken to ensure the same tightness if zip-ties are used to attach activity monitors to the collar.

The disconnect in previous publications [16, 18] between activity levels and other outcome measures such as gait analysis and owner questionnaires could be explained by the fact that activity levels represent a different measure of progression through a study period. While activity monitors may be addressing different aspects of outcome in these studies, the methods used to attach the activity monitors to the dogs cannot be ruled out as a source for the lack of correlation between activity levels and other measures utilized.

There were several limitations of this study, including the short duration that methods of attachment were evaluated for while walking. We chose this approach to eliminate variability in the type of activity, allowing for data acquisition in a controlled environment with a defined type of activity. Since activity monitors were directly compared for any given method of attachment, greater activity levels are likely only to further affect the results. Furthermore, given the large number of data points acquired, longer observation periods would be unlikely to change the results. It is also unknown how the varying activity levels of dogs in their daily lives would affect activity data. For instance, it is possible that the method of attachment would not have as large of an effect on a dog with a sedentary lifestyle. Further studies are necessary to characterize how the high and low extremes of activity level would impact activity data output amongst various methods of attachment. A second limitation of this study is that only one brand of accelerometer-based activity monitor was evaluated. Several other devices are available and it is unknown how the method of attachment would affect activity data from those monitors. However, until a similar evaluation of those devices is performed, we would suggest assuming that similar findings would apply to any activity monitor and the recommendations from this study should be followed regardless of which activity monitor is used. A final limitation of this study is the small sample size. However, in contrast to clinical studies evaluating naturally occurring disease, this study was designed to eliminate confounding factors and therefore a smaller sample size can be utilized to identify significant differences between groups. Additional studies with larger sample sizes that evaluate the identified factors in a clinical setting are necessary to further characterize accelerometer-based activity monitors as objective outcome measurement tools.

## Conclusions

In conclusion, when utilizing accelerometers as a research tool care must be taken to clearly specify the method of attachment. Since retrieving data from the activity monitor utilized in this study requires removal of the device from the collar, the method of attachment should be recorded and kept consistent throughout the study period. Connecting a leash to the collar to which an activity monitor is attached should be avoided, as it is difficult to keep the amount of tension on the lead while walking consistent throughout a study. Lastly, data obtained when using the protective case should not be compared to data collected without the casing. Our results indicate that the protective casing considerably affects the activity data. As such it may be advisable to avoid use of any case entirely.

## Abbreviations

CBPI: Canine Brief Pain Inventory
HCPI: Helsinki Chronic Pain Index

## Footnotes

1 Boots and Barkley^®^ Core Fashion Collar, Size Medium, Target Brands, Inc., Minneapolis, MN

2 Actical, Mini Mitter Company, Inc., Bend, Oregon

3 Easy Walk Harness, PetSafe^®^, Radio Systems Corporation, Knoxville, Tennessee

4 ActiReader, Mini Mitter Company, Inc., Bend, Oregon

5 Excel, Microsoft Corporation, Redmond, Washington

6 SPSS 22, IBM, Inc.
